# Systematic Review of Intravenous Ascorbate in Cancer Clinical Trials

**DOI:** 10.3390/antiox7070089

**Published:** 2018-07-12

**Authors:** Gina Nauman, Javaughn Corey Gray, Rose Parkinson, Mark Levine, Channing J. Paller

**Affiliations:** 1 National Institutes of Health, National Institute of Diabetes and Digestive and Kidney Diseases, Clinical Nutrition Section, Bethesda, MD 20892, USA; gn157@georgetown.edu (G.N.); markl@bdg8.niddk.nih.gov (M.L.); 2 Sidney Kimmel Comprehensive Cancer Center, Johns Hopkins Medical Institutions, Baltimore, MD 21287, USA; corey.gray@jhmi.edu (J.C.G.); rparkin1@jhmi.edu (R.P.)

**Keywords:** intravenous, ascorbate, vitamin C, clinical trials, cancer, patients

## Abstract

Background: Ascorbate (vitamin C) has been evaluated as a potential treatment for cancer as an independent agent and in combination with standard chemotherapies. This review assesses the evidence for safety and clinical effectiveness of intravenous (IV) ascorbate in treating various types of cancer. Methods: Single arm and randomized Phase I/II trials were included in this review. The PubMed, MEDLINE, and Cochrane databases were searched. Results were screened by three of the authors (GN, RP, and CJP) to determine if they met inclusion criteria, and then summarized using a narrative approach. Results: A total of 23 trials involving 385 patients met the inclusion criteria. Only one trial, in ovarian cancer, randomized patients to receive vitamin C or standard of care (chemotherapy). That trial reported an 8.75 month increase in progression-free survival (PFS) and an improved trend in overall survival (OS) in the vitamin C treated arm. Conclusion: Overall, vitamin C has been shown to be safe in nearly all patient populations, alone and in combination with chemotherapies. The promising results support the need for randomized placebo-controlled trials such as the ongoing placebo-controlled trials of vitamin C and chemotherapy in prostate cancer.

## 1. Introduction

Ascorbate (vitamin C) was proposed to have anticancer effects as early as the 1950s [1,2] However, the earliest effort to using high-dose vitamin C—both intravenously (IV) and orally—as a cancer treatment occurred in the 1970s, by Scottish surgeon Ewan Cameron and his colleague Allan Campbell. For comparison purposes, in 1974, the recommended dietary allowance of vitamin C was 0.045 g (45 mg) daily [3]. Cameron and Campbell treated 50 patients with various types of advanced cancers with high doses of oral ascorbate, IV ascorbate, or both. Several responses were observed following this treatment [4,5,6]. These findings led to a collaboration between Cameron and Nobel Prize winning chemist Linus Pauling on the evaluation of two case series of cancer patients [5,7]. The data obtained from these cancer patients suggested that there was a potential survival benefit when their treatment was supplemented with oral and IV vitamin C [7,8]. Limitations of these findings have subsequently been described [9], including that the findings were retrospective, without controls or blinding, and that studied patients may have been at risk for endemic vitamin C deficiency.

To test ascorbate prospectively, two randomized, placebo-controlled prospective trials were conducted at the Mayo Clinic, in which cancer patients received either placebo or 10 g of oral ascorbate. Each study noted no significant difference between the ascorbate-treated and placebo-treated groups [2,10]. Based on these results, ascorbates role in cancer treatment was dismissed [11,12]. However, there was renewed interest in the use of vitamin C as a cancer treatment, based on the discovery that intravenous ascorbate produced plasma ascorbate concentrations that were much higher than those from oral ascorbate, and were not possible from oral ascorbate [9,13,14]. Although Cameron’s subjects received both intravenous and oral ascorbate, subjects in the two randomized placebo-controlled trials at Mayo Clinic received only oral ascorbate. The significance of this key difference was not previously recognized until ascorbate pharmacokinetic studies in healthy subjects revealed the importance of the route of administration.

Subsequently, emerging preclinical and clinical studies led to a revival of interest into the clinical potential of intravenous ascorbate as a cancer chemotherapeutic agent, specifically its synergy with chemotherapy and amelioration of chemotherapy-induced side effects [15]. Additional studies on the efficacy of vitamin C as a therapeutic have shown that intravenous administration achieves high plasma concentrations that are not achievable through oral administration [13,16,17,18]. Specifically, oral administration of vitamin C at a dose of 1.25 g achieved a maximum plasma concentration of 134.8 ± 20.6 µmol/L (µM), while IV administration of vitamin C achieved a maximum plasma concentration of 885 ± 201.2 µmol/L [13,16]. In the text that follows, we refer to plasma ascorbate concentrations as pharmacologic when they can only be achieved by intravenous administration in humans, and as parenteral (intravenous or intraperitoneal) administration in rodents.

The role of intravenous vitamin C in combination with chemotherapy as a cancer treatment is still being examined and various trials into this subject matter are ongoing. This systematic review summarizes the clinical trials of IV ascorbate to date which were primarily composed of single-arm trials examining dose-limiting toxicities, progression-free survival, and overall survival.

### 1.1. Clinical Pharmacokinetics of Vitamin C

Clinical data show that intravenous and oral administration of ascorbate yield differing plasma concentrations. When ascorbate is given orally, fasting plasma concentrations are maintained at <100 μM [13] but when oral doses exceed 200 mg, the percentage of the absorbed dose decreases, with a decrease in ascorbate bioavailability, and renal excretion increases [13,19]. In contrast, intravenous administration bypasses the intestinal absorption system. This allows plasma concentrations to be elevated to pharmacologic concentrations (mmol/L [mM] values) that are unachievable via oral administration [20]. In healthy humans, plasma vitamin C concentrations were significantly higher following IV administration compared to oral dosing, and the difference in plasma concentration increased according to the dose delivered. It was found that the mean peak values from IV administration were 6.6-fold higher than the mean peak values from oral administration at a dose of 1.25 g vitamin C [16]. IV ascorbate can be administered by either bolus or continuous infusion. Bolus infusion can be considered as dosing based on pharmacokinetics that occurs over a defined period of time, usually 1.5–2 h [17,21,22]. Continuous infusion is usually considered over periods of time >12 h. Bolus administration of ascorbate has been used more commonly than continuous administration. With a dose of 1 g/kg, bolus administration produces peak plasma ascorbate concentrations of approximately 25 mM, with concentrations maintained above 10 mM for approximately 4 h and return to baseline (<0.1 mM) after approximately 12 h [18]. Following IV administration of pharmacologic ascorbate doses, the plasma half-life is as rapid as 0.5–1 h. With 10 g administered continuously over 24 h, steady-state plasma concentrations can be estimated to be approximately 1–2 mM [19,23]. When oral ascorbate intake stops, the plasma half-life is approximately 8–20 days, due to the action of renal transporters reabsorbing filtered ascorbate [9,18,24,25].

Additionally, some but not all preclinical data indicate that ascorbate can accumulate in solid tumors at higher concentrations than surrounding normal tissue [26,27,28]. This suggests that cancerous cells are especially affected by vitamin C, which favors the clinical potential of high-dose intravenous vitamin C as a cancer therapeutic [20].

### 1.2. Possible Mechanisms of Anti-Tumor Effects of Vitamin C

Several major mechanisms have been proposed to explain why only pharmacologic ascorbate concentrations have cytotoxic effects on some but not all cancer cells. Two mechanisms include increased pro-oxidant damage that is irreparable by tumor cells, and oxidation of ascorbate into dehydroascorbic acid (DHA), which is an unstable metabolite and can be cytotoxic [20]. Most data indicate that the first pathway predominates, specifically by generation of extracellular hydrogen peroxide (H_2_O_2_) by pharmacologic ascorbate and a trace transition metal, usually iron [29,30]. Hydrogen peroxide is cell permeant, and, in the presence of pharmacologic ascorbate, H_2_O_2_ reactive oxygen species (ROS) are formed extracellularly and/or intracellularly [31]. These ROS have multiple downstream targets, including but not limited to DNA damage, mitochondrial damage, and stimulation of apoptotic pathways [29,32,33].

To learn experimentally whether extracellular H_2_O_2_ is essential, the enzyme catalase is added. At concentrations used by nearly all laboratories, catalase is a non-permeant protein that dismutates H_2_O_2_ to water and oxygen. The great majority of in vitro work shows that cell death is blunted or eliminated by catalase addition, pointing to the key role of H_2_O_2_. The second pathway involves dehydroascorbic acid (DHA), the reversible oxidized form of ascorbate. This pathway is based on findings that tumor cells transport DHA and then internally reduce it to ascorbate. In specifically engineered cells, this reduction triggers scavenging of glutathione (GSH), induces oxidative stress, inactivates glyceraldehyde 3-phosphate dehydrogenase (GAPDH), inhibits glycolytic flux, and leads to an energy crisis that triggers cell death [34,35]. DHA findings are attractive, but have several limitations, including that extracellular H_2_O_2_ may still be the initial driver of ascorbate oxidation to DHA, and that DHA does not cause cell death in a variety of unmodified cancer cells that do respond to ascorbate [29,30,36,37].

Two additional mechanisms of ascorbate action in cancer are based on ascorbate’s activity as a cofactor for Fe (II) 2-oxoglutarate dioxygenase enzymes. As a co-factor, ascorbate modulates DNA demethylation and epigenetic marks through interaction with the ten eleven translocation (TET) enzyme family [38,39]. Ascorbate binds to the catalytic domain facilitating TET-mediated DNA demethylation [38,40]. This reverses the hypermethylation triggered in oncogenic states and subsequently activates tumor suppressor genes [40,41]. Reactivation of tumor suppressor genes allows for anti-tumor mechanisms to become active and increases chemosensitivity. Ascorbate action on TET may have promise in preventing tumor development especially in myelodysplastic syndrome [3,42]. Similarly, ascorbate acts as a co-factor for hypoxia-inducible transcription factors (HIFs) prolyl-4-hydroxylase domain (PHD) enzymes. Prolyl-4-hydroxylation is necessary for targeting of HIFs for proteolytic degradation [43,44,45]. In solid tumors, HIF-1 helps tumor cells shift from aerobic metabolism to anaerobic metabolism increasing flux through glycolysis to maintain energy production [43]. This activity in tumor cells creates a state that is dependent on glycolytic metabolites. It is possible that the DHA mechanism discussed above works in tandem with the HIF mechanism to cause global disruption of metabolic functioning in the tumor cell triggering cell death. For both TET-mediated and HIF-mediated mechanisms, ascorbate action at physiologically relevant concentrations may prevent cancer development. For cancer treatment, only pharmacologic ascorbate was found to be effective [30].

For the majority of cancer cells in vitro, ascorbate concentrations less than 5 mM are sufficient to induce a 50% decrease in cell survival. In contrast, many non-cancerous cells are capable of tolerating ascorbate concentrations of 20 mM, indicating less sensitivity [36]. Note that in vitro there is some heterogeneity in response to ascorbate in tumor and non-tumor cells alike. Perhaps 10–15% of cancer cells are insensitive to 20 mM ascorbate. Moreover, the death of cancer cells is thought to be selectively induced by extracellular ascorbate, and not intracellular ascorbate [17,36,46,47].

### 1.3. Synergy with Chemotherapy

Translational synergy of pharmacologic ascorbate with chemotherapy was first demonstrated using cell and mouse pancreatic cancer models [48]. Ascorbate was synergistic with gemcitabine both in vitro and in vivo, without apparent harm. The synergy of ascorbate with conventional chemotherapy is the subject of many clinical studies (Table 1 and Table 2). Further, ascorbate was permissive for dose reductions of gemcitabine in these pre-clinical studies. These findings have clinical promise, but to date only individual cases have been reported, without data for failure rates [49]. Ascorbate synergy with conventional chemotherapy was also rigorously investigated in ovarian cancer models. The combination of ascorbic acid and conventional chemotherapeutic agents synergistically inhibited ovarian cancer cell lines and xenografts in mice [50]. Ma et al. exposed ovarian cancer cell lines (OVCAR5, OVCAR8, and SHIN3) to ascorbate and carboplatin in varying molar ratios, using HIO-80 cells, a nontumorigenic ovarian cell line, as a control. The results of this preclinical study demonstrated that the combination of ascorbate and carboplatin induced greater cell death in all cancer cell lines compared to either drug individually [50]. The HIO-80 ovarian epithelial cell line was shown to be equally sensitive to carboplatin alone, and the ascorbate-carboplatin combination. The SHIN3 cell line was implanted into athymic mice to further test the synergistic effect. Ascorbate and carboplatin were shown to be more effective at reducing tumor burden compared to either ascorbate or carboplatin alone. Clinically, multiple trials have demonstrated the safety of ascorbic acid when combined with chemotherapy in the treatment of several cancers including multiple myeloma, ovarian and pancreatic cancer [21,50,51,52].

## 2. Materials and Methods

This review’s protocol was developed by the authors and was designed to summarize the results of clinical trials in which cancer patients are treated with intravenous vitamin C, either as a single agent or in combination with standard therapies. The population of interest for this review included patients with a current diagnosis of cancer of any type and stage. The intervention of interest was treatment with intravenous ascorbate alone or in combination with standard cancer therapies. Uncontrolled studies or controlled studies that included comparisons against no treatment, placebo, or other standard of care therapies were of interest. Outcomes of interest included Common Terminology Criteria for Adverse Events (CTCAE) adverse events or other measured toxicities, quality of life, progression free survival and overall survival. Randomized controlled trials were of primary interest, but all study designs were included in the initial search.

**Figure 1:**
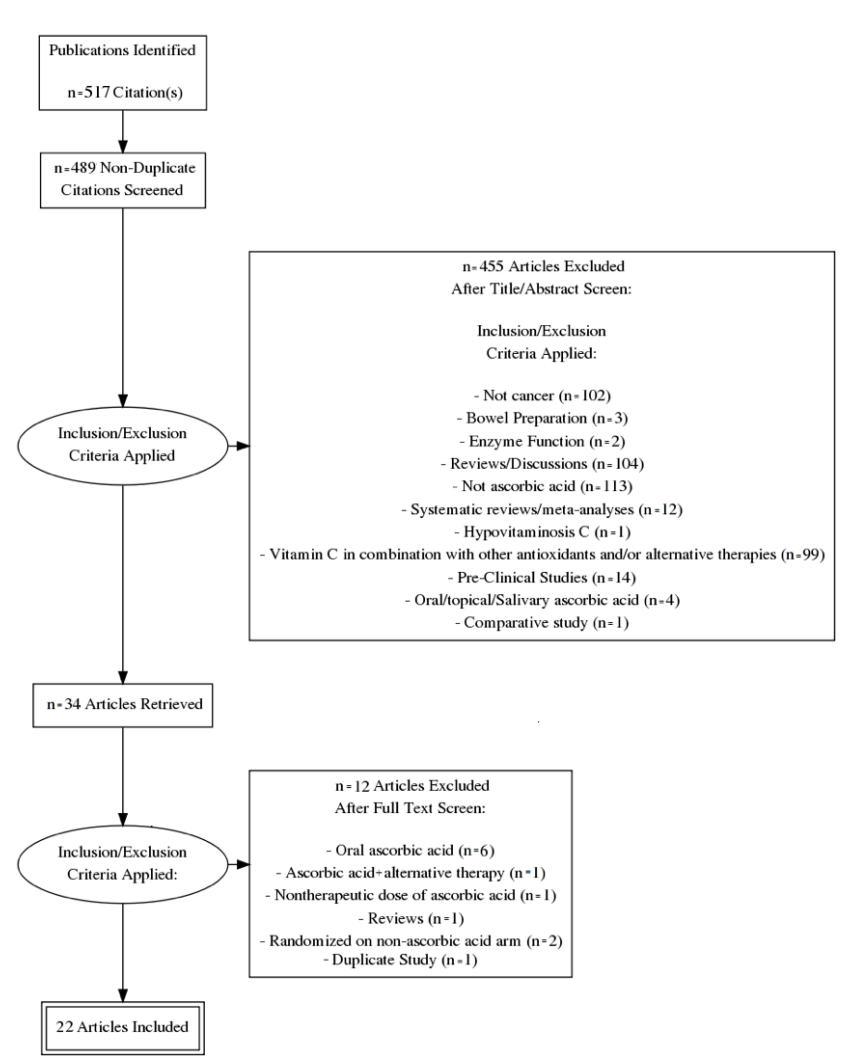
Prisma Flow Diagram.

An electronic literature search was conducted in the PubMed, MEDLINE, and Cochrane databases. PubMed served as an interface for searching MEDLINE (Figure 1). The exact search term combination used in the PubMed search was: “Ascorbate OR Vitamin C AND Cancer NOT Bowel Preparation AND Clinical Trial”. The exact search term combination used in the Cochrane database search was: “cancer” “vitamin c” “clinical trial”. Following retrieval of the studies, three authors screened the studies (GN, RP, and CJP), eliminated duplicates, and removed all studies that were not clinical trials or not relevant to the subject matter. These authors then screened the remaining studies a second time, removing trials that examined oral ascorbate, and trials that were terminated prematurely and/or had no results. After the second screening, the remaining studies were summarized using a narrative approach.

### Study Characteristics

A total of 22 articles (containing 23 trials) that included 401 patients evaluated IV ascorbate (Table 1, Table 2 and Table 3). Of these trials, eleven trials evaluated arsenic trioxide in combination with intravenous ascorbic acid clinical trials, nine evaluated intravenous ascorbic acid in combination with non-redox cycling agents, and three trials evaluated intravenous ascorbic acid alone. The median sample size of these studies was 17 (range, 3–65) and the IV dose of ascorbate ranged from 1 g daily to 1.5 g/kg thrice weekly.

## 3. Results

### 3.1. Trials Evaluating Low-Dose Intravenous Ascorbic Acid in Combination with Arsenic Trioxide

Out of the 23 clinical trials included in this paper, 11 trials with a total of 200 patients used low dose intravenous ascorbic acid in combination with arsenic trioxide (As_2_O_3_) (Table 1). In the study design of trials such as these, ascorbic acid does not act as an anti-cancer therapy in its own right. The dose used in such studies (one gram per day) is not considered a pharmacologic or effective dose. The justification for selecting this dose is that when given orally it saturates plasma and ascorbate tissue ascorbate concentrations. However, in most studies, ascorbate was administered intravenously, for unclear reasons [29]. Ascorbate was added as a redox cycling compound to facilitate the anti-cancer activity of As_2_O_3_. Thus, for these trials, the main source of anti-cancer activity is As_2_O_3_ and all of the effects produced in these trials should be attributed to As_2_O_3_ [29].

Berenson et al. reported two phase II trials [52,53] in patients with refractory/multiple myeloma that included low dose IV ascorbic acid. In the 2006 study, patients (*n* = 65) received IV ascorbic acid (1 g on Days 1–4) and As_2_O_3_ and melphalan. A response rate of 48% was observed with a progression-free survival of seven months and overall survival of 19 months [53]. In the 2007 study, patients (*n* = 22) were treated with IV ascorbate (1 g on Days 1, 4, 8, and 11 of a 21-day cycle for a maximum of eight cycles) in combination with Bortezomib and As_2_O_3_ [52]. A response rate of 27% was observed and median progression-free survival was five months. Both studies reported grade 3/4 adverse events. Because of the trial design and types of toxicities, adverse events were likely related to chemotherapy.

Two As_2_O_3_ trials examined the benefits of IV ascorbate in combination with chemotherapy used response rate as the primary outcome [54,55]. Abou-Jawade et al. reported a single arm study of IV ascorbate (1000 mg daily for five days and then twice weekly for nine weeks) in combination with Dexamethasone and As_2_O_3_ for patients of relapsed and refractory myeloma (*n* = 20). The authors reported an overall response rate of 30%, which included both partial and complete response. Ten patients developed grade 3 or 4 toxicity to this treatment combination, although toxicity due to ascorbate was not defined. Chang et al. reported a similar phase II trial in which patients with lymphoid malignancies (*n* = 17) were treated with IV ascorbate (1000 mg daily for five days then twice weekly) alongside As_2_O_3_. An overall response rate of 6% was reported and severe toxicities (multiple grade 3, 4, and 5 events) were observed. The trial was closed after the first interim analysis due to lack of activity. Similarly, in a phase II trial by Bael et al. in patients with advanced melanoma (*n* = 11) being treated with IV ascorbate (1000 mg for five days for one week, then twice weekly for an additional eight weeks) in combination with Temozolomide and As_2_O_3_, no responses were seen in the first 10 evaluable patients leading to early closure of the study [56].

Three trials examined the benefit of IV ascorbate (1000 mg/day for five days) in combination with As_2_O_3_ only [57,58,59]. Subbarayan et al. reported a study in which patients of refractory metastatic colorectal carcinoma (*n* = 5) were treated with this combination, and multiple grade 3 events were reported (nausea, vomiting, diarrhea, thrombocytopenia, and anemia), although no complete or partial remission was observed [57]. Wu et al. reported a similar trial with patients of relapsed/refractory multiple myeloma (*n* = 20), but this study was reported in a letter format only [58]. A median survival time of 11 months was observed. In the 2014 study by Aldoss et al., however, intravenous AA was evaluated in combination with As_2_O_3_, which is highly effective in acute promyelocytic leukemia (APL), but, despite its multiple mechanisms of action, it has no activity in acute myeloid leukemia (AML) that excludes APL (non-APL AML). The patient population (*n* = 11) in this study were all diagnosed with non-APL AML and were administered intravenous As_2_O_3_ (0.25 mg/kg/day over 1–4 h) with intravenous AA (1 g/day over 30 min after As_2_O_3_) for five days a week for five weeks (25 doses). Among 10 evaluable patients, one achieved a complete response, one achieved a partial remission with incomplete hematologic recovery, and four patients had disappearance of blasts from peripheral blood and bone marrow. The observed As_2_O_3_ toxicity was mild; very few grade 3 or 4 adverse effects and the most common grade 3 toxicity was infection, although possibly related to the leukemia. The authors concluded that combination of As_2_O_3_ and AA had limited clinical meaningful anti-leukemia activity in patients with non-APL AML [59].

Bahlis et al. reported a study using As_2_O_3_ in combination with IV ascorbate to ascertain dosing of As_2_O_3_. Patients of refractory myeloma (*n* = 6) were treated with IV ascorbate dose of 1000 mg/day for 25 days over 35 days total, and 0.25 mg/kg per day of As_2_O_3_ was defined as an appropriate dose [60]. A partial response rate of 36% was observed and no toxicities above grade 2 were reported. It is unclear if these toxicities were due to the addition of ascorbic acid, increased As_2_O_3_, the schedule, or duration of treatment. Held et al. reported a similar phase I trial that also aimed to estimate the maximum tolerated dose of As_2_O_3_ and bortezomib that can be used in combination with IV ascorbate (1 g daily for three days of Week 1, then twice weekly for a three-week cycle) in patients with relapsed/refractory multiple myeloma (*n* = 10) [61]. No dose-limiting toxicities were reported and a 40% response rate was reported. Welch et al. reported a trial with patients of myelodysplastic syndrome and acute myeloid leukemia (*n* = 13) being treated with Decitabine and As_2_O_3_ and IV ascorbate (1000 mg for five days during Week 1 following each dose of IV As_2_O_3_ and then once weekly thereafter) [62]. Five patients had stable disease after recovery and multiple grade 3 and 4 events were reported; the authors stated that these adverse events were expected given the patient population and type of chemotherapy but did not clarify if the addition of ascorbate was a contributing factor.

### 3.2. Trials Evaluating High-Dose Intravenous Ascorbic Acid with Standard Chemo- and Radiotherapy Agents

Stephenson et al. reported a phase I trial with patients with advanced malignancies (*n* = 17) being treated with IV ascorbate 70–80 g/m^2^ (this translates to approximately 125 g because the average patient has body surface of 1.6–1.9 m²) [67] 3–4 times a week to obtain optimal peak plasma concentrations in combination with multivitamin and eicosapentaenoic acid treatment [63]. Only two patients completed the entire four-week study period, and stable disease rate and progressive disease rate of 19% and 81% were reported, respectively. Grade 3 and 4 metabolic toxicities (hypernatremia and hypokalemia) related to ascorbate was observed. Kawada et al. reported a similar study in patients with relapsed lymphoma (*n* = 3) that were treated with rituximab, cyclophosphamide, cytarabine, etoposide, and dexamethasone alongside IV ascorbate (75 g twice weekly) [64]. Grade 3 neutropenia, anemia, and thrombocytopenia were observed, but no obvious side effects due to ascorbic acid were observed, leading the authors to conclude that 75 g of IV ascorbate is a safe dose. It is likely that hypernatremia and hyperkalemia, reported by Stephenson et al., was secondary to the approximately two-fold higher ascorbate dose that patients received in comparison to other trials.

Two phase I trials examined the benefits of IV ascorbate in combination with gemcitabine in patients with advanced pancreas adenocarcinoma [21,51]. Both Welsh et al. (*n* = 13) and Monti et al. (*n* = 14) reported toxicity in patients related to gemcitabine and not secondary to ascorbate [21,51]. Response rates and survival duration in both studies were reported only for patients who did not progress within the first month of treatment and are thus not representative of standard clinical reporting. Monti et al. reported that seven of nine patients had stable disease with a mean overall survival of 155 ± 182 days and Welsh et al. reported a 13 ± 2-month mean survival in the nine patients that were analyzed.

Ma et al. reported a trial of patients with stage 3 and 4 ovarian cancer (*n* = 25) receiving carboplatin and paclitaxel chemotherapy [50]. Patients were randomized to either IV ascorbate (75 g or 100 g twice weekly for 12 months) with chemotherapy (*n* = 13) or chemotherapy alone (*n* = 12). The trial was not blinded and the primary outcome was toxicity. The ascorbate group was observed to have fewer grade 1/2 adverse events per encounter as compared to the group that received only chemotherapy. A trend toward improvement in median overall-survival was reported, although no numerical data were reported. Median time for disease progression/relapse was reported as 25.5 months in the ascorbate arm and 16.75 in the chemotherapy arm. This trial also demonstrated key information related to the safety profile of ascorbate as patients were treated for more than a year with minimal adverse effects.

Hoffer et al. (2015) reported a study with patients of various cancer types (*n* = 16) treated with IV ascorbate. Patients were administered ascorbate at a dose of 1.5 g/kg three times on weekdays during weeks when chemotherapy was administered, and at least one day apart during weeks when no chemotherapy was given). This was given in combination with standard care chemotherapy, which was not defined [22]. Adverse effects included increased thirst and urinary flow. Transient stable disease, increased energy, and functional improvement were observed in patients.

Schoenfeld et al. (2017) reported a phase I study with glioblastoma (GBM) patients (*n* = 13) receiving pharmacological ascorbate with radiation and temozolomide [30]. The study had two phases: the radiation phase (which started on Day 1 of the radiation phase and ended on Cycle 1, Day 1 of the adjuvant period) and the adjuvant phase (which began on Cycle 1, Day 1 until Cycle 6, Day 28) [30]. The participants in the radiation phase received radiation (61.2 Gy in 34 fractions), temozolomide (75 mg/m^2^ daily for a maximum of 49 days) and ascorbate (dose cohorts ranging from 15–125 g, three times per week for seven weeks) [30]. In the adjuvant phase, participants received temozolomide (Days 1–5 of a 28-day cycle and one dose-escalation to 200 mg/m^2^ took place if Cycle 1 was tolerable) and ascorbate (infusions took place two times per week and dose was increased over two infusions until a plasma concentration of 20 mM was reached, which was achieved with an 87.5 g infusion) for about 28 weeks [30]. Adverse effects in the radiation phase included grade 2 and 3 fatigue and nausea, grade 2 infection, and grade 3 vomiting. In the adjuvant phase, patients experienced grade 2 fatigue and nausea, grade 1 vomiting, grade 3 leukopenia, and neutropenia. At the time of publishing in 2017, the average PFS with Schoenfeld et al.’s therapy was 13.3 months as compared to PFS of seven months in Stupp et al. (2005) [61] which treated GBM patients with similar characteristics with concurrent radiation and temozolomide or radiation only. Average overall survival was 21.5 months as compared to 14 months in Stupp et al., 2005 [30]. 

Schoenfeld et al. (2017) also reported a phase II study with advanced stage non-small-cell-lung carcinoma (NSCLC) patients (*n* = 14) treated with carboplatin, paclitaxel and pharmacological ascorbate [30]. Participants were administered IV carboplatin (AUC 6, four cycles), IV paclitaxel (200 mg/m^2^, four cycles), and IV pharmacological ascorbate (75 g per infusion, two infusions per week, up to four cycles); one cycle was 28 days [30]. No grade 3 or 4 toxicities related to ascorbate were noted. Imaging-confirmed partial responses to therapy in patients who completed the trial (*n* = 4), stable disease (*n* = 9), and new lesion development (*n* = 1) indicating disease progression despite the patient having a stable target lesion [30].

Polireddy et al. (2017) reported a Phase I/IIa trial with locally advanced or metastatic prostate cancer patients (*n* = 14) who were not eligible for surgical resection with high-dose IVC and gemcitabine chemotherapy [62]. Phase I initially enrolled 14 patients but only 12 patients completed a pharmacokinetic evaluation of IVC and gemcitabine alone. IVC dose escalated from 25 g to 100 g and gemcitabine dose at 1000 mg/m^2^, with a few patients receiving reduced doses as determined by the treating oncologist give from Week 1 to Week 4 and subsequently in combination during Week 4. In Phase IIa, the 12 patients were given IVC three-times weekly at doses determined by the treating oncologist and gemcitabine following a rest week after two consecutive weeks of a determined dose and then treatment until tumor progression or patient withdrawal. Overall survival was 15.1 months with 5 of 12 patients not surviving over one year, 6 of 12 patients surviving over one year, and 1 of 12 surviving more than two decades after diagnosis. Over the course of treatment, one patient with Stage III pancreatic ductal carcinoma experienced tumor shrinkage/stabilization and tumor margins becoming more distinct, making the patient eligible for surgery. Grade 1 nausea and thirst related to IVC were the only adverse events noted. The study showed IVC has low toxicity and does not alter gemcitabine pharmacokinetics significantly.

### 3.3. IV Ascorbate Only Trials

Hoffer et al. (2008) reported a phase I trial in patients with advanced cancer or hematologic malignancy (*n* = 24) treated with up to 1.5 g/kg body weight of IV ascorbate three times weekly. No dose limiting adverse effects were reported and two patients had unexpectedly stable disease [18]. Nielsen et al. (2017) reported a similar study in patients with castration-resistant prostate cancer (*n* = 23) treated with IV ascorbate 5 g once during Week 1, 30 g weekly during Week 2, and 60 g once weekly during Weeks 3–12 [34]. Multiple grade 3 events were reported including hypertension and anemia; two patients experienced a pulmonary embolism; however, the authors stated that treatment-induced toxicity was limited and the two episodes of pulmonary embolism can likely be attributed to the fact that cancer is known to increase the risk of thromboembolic events. However, without a placebo-controlled trial, attribution to disease or ascorbate cannot be definitely proven. Both studies reported no anticancer response or disease remission.

Lastly, Riordan et al. reported a trial in which late stage terminal cancer patients (*n* = 11) were given continuous infusions of 150–710 mg/kg/day for up to eight weeks. Intravenous infusions increased plasma ascorbate concentrations to a mean of 1.1 mM. Two Grade 3 adverse events to the agent were reported: one patient with a history of renal calculi developed a kidney stone after thirteen days of treatment and another patient experienced hypokalemia after six weeks of treatment; the authors state that these adverse events could possibly be related to ascorbic acid, but it remains unclear. One patient had stable disease and continued the treatment for forty-eight weeks. The authors concluded that intravenous vitamin C administered continuously is relatively safe so long as the patient does not have a history of kidney stone formation [23].

### 3.4. Potential of Benefit and Current Limitations

Clinical trials that have examined the use of IV ascorbate in cancer patient populations have yielded results that suggest its potential to produce various beneficial effects. In one trial, IV ascorbate was used in elderly patients with advanced cancer who had failed all other therapies. Two of the patients had unexpected stable disease after eight weeks of ascorbic acid treatment [18]. A phase I trial in patients with metastatic stage 4 pancreatic cancer who were treated with gemcitabine and IV ascorbate as primary therapy until tumor progression showed few toxicities associated with the treatment [51]. The nine patients had a tripling of disease free interval compared to literature controls and a doubling of survival compared to retrospective controls. Some patients were treated for longer durations, for instance over a year in the Ma et al. trial [50], and had substantially decreased grade 1 and 2 adverse events when compared to the group not receiving ascorbate.

Similarly, a trial of patients with metastatic stage 4 pancreatic cancer illustrated benefit as eight of nine patients experienced tumor shrinkage after eight weeks of primary therapy (gemcitabine and erlotinib) and pharmacological ascorbate as measure by CT scans [21]. The results of these trials and others discussed in this article suggest that IV ascorbate is useful as a single agent or combined with a primary therapy. It has the benefit of being a non-toxic treatment modality and reducing toxicity of chemotherapeutics when combined with conventional therapies. In one randomized ovarian cancer trial, patients receiving IV ascorbic acid reported lower levels of low-grade gastrointestinal, hepatobiliary, dermatological, immune/infection, pulmonary and renal toxicities commonly associated with carboplatin and paclitaxel treatment [50]. One retrospective cohort study [68] compared breast cancer patients who received IV ascorbic acid to those who did not, at a dose of 7.5 g weekly without blinding. In the first year following surgery, patients who received ascorbate when compared to a control group had significant reductions in nausea (*p* = 0.022), loss of appetite (*p* = 0.005), fatigue (*p* = 0.023), dizziness (*p* = 0.004) and hemorrhagic diathesis (*p* = 0.032). Limitations of this study are the absence of blinding, the non-therapeutic dosing and once weekly frequency of ascorbate administration. Even so, the results suggest that IV ascorbate could induce reduction in toxicities, perhaps via mechanisms that are different than those that target cancer cells. To definitively associate IV ascorbate with clinical benefit and/or toxicity, more rigorous randomized-placebo trials must be conducted. Many of the clinical studies conducted to date do not contain a control group, which makes determining efficacy difficult. 

In addition to examining efficacy, there is need for a determination of IV AA dosing amount, dosing frequency, and duration of treatment alone or in combination with other therapies. Currently, there is no consensus on these parameters. For dosing of intravenous ascorbate, doses used most frequently are based on one of a few regimens suggested by Riordan and colleagues. The goal was to achieve a plasma concentration of approximately 22 mM, which was effective in a hollow-fiber tumor model [69]. This dosing amount translates as approximately 1 g/kg. For dosing frequency, a regimen of 2–3 times weekly was empiric, with patient ability and/or willingness to receive treatment being limiting factors. Considering dosing amount and frequency together, therapy less than twice weekly, with dosing less than 1 g/kg, appears to be therapeutically ineffective [66], while dosing at 1 g/kg at least twice weekly has promise [21,51]. For duration needed to assess responsiveness, clinically detectable ascorbate action is relatively slow compared to many other cancer therapies. In most reports, a minimum 2–3-month time frame was needed to assess response [17,21,51,69,70]. Due to unknowns about concomitant administration with standard chemotherapies, ascorbate most often has been administered alone, without other chemotherapy on the same day. When ascorbate was administered on the same day as chemotherapy in a series of cases, the clinical response was seemingly enhanced by ascorbate [66,70]. Unfortunately, in this case series, only minimal information was provided about adverse events and non-responders. Based on the totality of available evidence and our experience, some recommendations can be made for future studies. These recommendations include that ascorbate dosing should be 1 g/kg, at a minimum frequency of twice weekly, and with a minimum of a two-month and preferably three-month trial period before efficacy is assessed. However, further research into the potential benefit of IV AA is necessary before well-defined clinical recommendations can be made.

## 4. Future Directions

**Figure 2:**
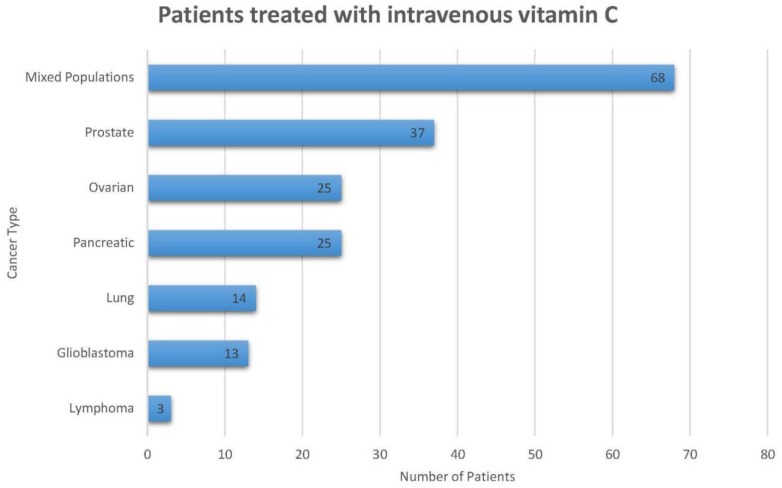
This figure represents the total number of cancer patients (*n* = 185) that were treated with intravenous ascorbic acid within the clinical trials summarized in this paper. This figure does not include patients who were enrolled in trials that used arsenic trioxide.

Thus far, a total of 185 cancer patients have been treated with IV vitamin C within the clinical trials discussed in this review, excluding those treated with low-dose vitamin C (redox coupling mechanism) (Figure 2). Moreover, there are 11 studies in progress that aim to investigate the clinical efficacy of pharmacological IV ascorbate (Table 4) including a total of 405 patients. There are two randomized studies (NCT03175341 and NCT02516670) and two non-randomized studies (NCT01852890 and NCT01752491). Both hematological and solid organ malignancies are being evaluated. Note that, even though all studies using ascorbate are included, ascorbate dosing may be well below that considered pharmacologic dosing.

In the Phase I trials, the safety of high dose ascorbate is being tested in combination with gemcitabine and radiation therapy (NCT01852890), temozolomide, and radiation therapy (NCT01752491), and gemcitabine, cisplatin, and nab-paclitaxel (NCT03410030). These studies aim to further determine the safety and toxicity of high dose ascorbate, in addition to establishing a pharmacokinetic profile, elucidating the biological responsiveness, and determining its efficacy in reducing side effects of chemotherapy.

In the Phase II trials, ascorbate is being tested in combination with gemcitabine and nab-paclitaxel (NCT02905578), temozolomide and radiation therapy (NCT02344355). The randomized Phase II trials, such as *Docetaxel with or without Ascorbic Acid in Treating Patients with Metastatic Prostate Cancer (NCT02516670)*, provide a vehicle for assessing the clinical effectiveness of ascorbate in a high-quality, placebo-controlled setting. These kinds of trials have the potential to further elucidate any synergistic anticancer effects that IV ascorbate might have when used in combination with chemotherapeutic agents. This has the potential to provide patients with additional treatment options.

In addition to these clinical studies, laboratory research has attempted to elucidate the mechanism of action of vitamin C in cancer cells. Yun et al. showed that high dose vitamin C selectively killed KRAS and BRAF mutants in colorectal cancer cells by inducing increased uptake of oxidized vitamin C and targeting the glycolytic pathway [34], although these findings have not been confirmed by others [30,36,48]. Nevertheless, potential remains to identify subtypes of cancer that might benefit from IV pharmacological ascorbate in a clinical setting.

## 5. Conclusions

Evidence supporting the existence of an anticancer effect of intravenous ascorbate is mixed, whether it is given a single agent or in combination with other concurrent standard therapies. In single-arm trials that used IV ascorbate in combination with concurrent standard therapies, it is unclear which agent delivered which effects. Only one randomized clinical trial has been reported, showing a trend toward overall survival, a significant 8.5 week increase in progression-free survival, and decreased adverse events in the IV ascorbate arm in ovarian cancer patients.

Current research indicates that IV ascorbate is well tolerated and has reported some positive results. However, high-quality placebo-controlled trials such as those being offered in prostate (NCT02516670) and breast cancer (NCT03175341) are needed to strengthen the present evidence base to support continuation of IV ascorbate being offered as a treatment by practitioners. Until these trials are completed, patients should be informed of the investigational status of IV ascorbate as a cancer treatment.

## Figures and Tables

**Table 1:** Low dose IV ascorbate + arsenic trioxide trials—Phase I and II trials.

Reference	*n*	Patient Diagnosis	Trial Design	IV AA Treatment Type and Frequency	Concurrent Treatment Dose	Toxicity	Reported Outcomes/Conclusions
[52]	22	Refractory multiple myeloma	Single Arm	1 g on days 1, 4, 8, and 11 of a 21-day cycle for a maximum of 8 cycles	Bortezomib and Arsenic Trioxide	One occurrence of grade 4 thrombocytopenia was observed in a patient receiving high-dose bortezomib	Objective responses were observed in 27% of patients (2 partial and 4 minor). Median progression-free survival was 5 months and overall survival had not been reached.
[53]	65	Relapsed or refractory multiple myeloma	Single Arm	1 g on days 1–4 of week 1 and twice weekly during weeks 2–5 of a 6 week cycle.	Melphalan and Arsenic Trioxide	Grade 3/4 hematological (3%) or cardiac adverse events occurred infrequently, but grade 3/4 adverse events fever/chills (15%), pain (8%), and fatigue (6%) were reported.	Objective responses occurred in 48% of patients, including complete, partial, and minor responses. Median progression-free survival and overall survival were 7 and 19 months respectively.
[54]	20	Multiple myeloma, relapsed and refractory	Single Arm	1000 mg for 5 consecutive days during week 1, followed by twice weekly during weeks 2–12	Dexamethasone and Arsenic Trioxide	Grade 3 events in 45% and grade 4 events in 5%	30% complete and partial response. Overall median survival was 962 days. 10 patients developed grade 3/4 toxicity to combination treatment.
[55]	17	Lymphoid malignancies, relapsed and refractory.	Single Arm	1000 mg for 5 days during week 1 followed by twice weekly during weeks 2–6	Arsenic Trioxide	1 cardiac death, multiple grade 3 and 4 events	Overall median survival was 7.6 months 6% complete and partial response. Study closed at first interim analysis.
[56]	11	Advanced melanoma	Single Arm	1000 mg for 5 days during week 0, and then twice weekly for an 8 week cycle.	Temozolomide and Arsenic Trioxide	Multiple grade 1 and 2 events.	No responses seen in the first 10 evaluable patients leading to early closure of study.
[57]	5	Refractory metastatic colorectal carcinoma	Single Arm	1000 mg/day for 5 days a week for 5 weeks	Arsenic Trioxide	Grade 3 nausea, vomiting, diarrhea, thrombocytopenia, and anemia	No complete or partial remission observed. CT scans showed stable or progressive disease.
[58]	20	Multiple myeloma, relapsed and refractory	Single Arm	1 mg (one dose during the first week, twice weekly during weeks 2–4)	Dexamethasone and Arsenic Trioxide	Multiple grade 3 and 4 events	Clinical response was observed in 40% of patients (including partial and minor). Median progression free survival = 4 months and median overall survival = 11 months. Authors state that it was difficult to assess activity of each individual agent.
[59]	11	Non-acute promyelocytic leukemia; acute myeloid leukemia (non-APL AML)	Single Arm	1 g/day for 5 days a week for 5 weeks	Arsenic Trioxide	Few grade 3 or 4 adverse effects and the most common grade 3 toxicity was infection though possibly related to the leukemia	One patient achieved a complete response; another achieved a complete remission with incomplete hematologic recovery. Authors concluded that arsenic trioxide + ascorbic acid had limited clinical meaning in non-APL AML patients.
[60]	6	Relapsed or refractory myeloma	Single Arm	1000 mg/day for 25 days over 35 days total.	Arsenic Trioxide	One episode of grade 3 hematologic toxicity (leukopenia) was observed.	Two patients had partial responses; four had stable disease.
[61]	10	Relapsed/refractory multiple myeloma	Single Arm	1 g daily for 3 days of week 1, then twice weekly for a 3-week cycle.	Arsenic Trioxide and Bortezomib	No dose limiting adverse effects.	40% response rate with one patient achieving a durable partial response.
[62]	13	Myelodysplastic Syndrome and Acute Myeloid Leukemia (concurrent diagnoses)	Single Arm	1 g for 5 days during week following each dose of IV Arsenic Trioxide and then once weekly thereafter	Decitabine and Arsenic Trioxide	Grade 3 and 4 events; two patient deaths occurred not related to treatment	One morphologic complete remission was observed. Five patients had stable disease after recovery. 0.2 mg/kg identified as maximum tolerated dose of arsenic in combination with Decitabine and Ascorbic Acid.

Note: This table illustrates the eleven clinical trials that evaluated intravenous ascorbate in combination with arsenic trioxide.

**Table 2:** High dose IV ascorbate + standard therapies—Phase I and II Trials.

Reference	*n*	Patient Diagnosis	Trial Design	IV AA Treatment Type and Frequency	Concurrent Treatment Dose	Toxicity	Reported Outcomes/Conclusions
[63]	17	Advanced tumors	Single Arm	Five cohorts treated with 30, 50, 70, 90, and 110 g/m^2^ for 4 consecutive days for 4 weeks.	Multivitamin and Eicosapentaenoic acid	Grade 3 and grade 4 hyponatremia, hyperkalemia	3 patients had stable disease, 13 had progressive disease. Recommended dose is 70–80 g/m^2^. This translates to approximately 125 g because the average patient has a body surface area of 1.6–1.9 m².
[64]	3	Relapsed lymphoma	Single Arm	75 g twice weekly	Rituximab, cyclophosphamide, cytarabine, etoposide, dexamethasone	Grade 3 neutropenia, anemia, thrombocytopenia	The authors concluded that 75 g was a safe dose.
[51]	11	Advanced pancreatic adenocarcinoma	Single Arm	15–125 g twice weekly	Gemcitabine	No dose limiting adverse effects	Mean plasma ascorbate levels were significantly higher than baseline. Mean survival time of subjects completing 8 weeks of therapy was 13 ± 2 months.
[21]	14	Pancreatic adenocarcinoma, stage IV	Single Arm	50, 75, and 100 g per infusion (3 cohorts) thrice weekly for 8 weeks	Gemcitabine and Erlotinib	Multiple toxicities, all grades, thought to not be related to AA; grade 4 adverse event included two patients with pulmonary embolism	50% of patients had stable disease. Survival analysis excluded 5 patients who progressed quickly (3 died). Overall mean survival was 182 days.
[50]	25	Stage 3/4 ovarian cancer	Randomized	75 or 100 g twice weekly for 12 months (target plasma concentration 20–23 mM)	Carboplatin and paclitaxel	Ascorbate did not increase grade 3/4; grade 1 and 2 toxicities were substantially decreased	8.75 month increase in PFS in AA-treated arm. Trend to improved OS in AA group; no numerical data reported.
[22]	16	Various cancer types (lung, rectum, colon, bladder, ovary, cervix, tonsil, breast, biliary tract)	Single Arm	1.5 g/kg body weight infused three times (at least one day apart) on week days during weeks when chemotherapy was administered (but not on the same day as intravenous chemotherapy) and any two days at least one day apart during weeks when no chemotherapy was given.	Standard care chemotherapy.	Increased thirst and increased urinary flow; these adverse symptoms did not appear to be caused by the ascorbate molecule	Patients experienced unexpected transient stable disease, increased energy, and functional improvement.
[30] Phase I study	13	Glioblastoma	Single Arm	Radiation phase: radiation (61.2 Gy in 34 fractions), temozolomide (75 mg/m^2^ daily for a maximum of 49 days), ascorbate (doses ranging from 15–125 g, 3 times per week for 7 weeks) Adjuvant phase: 6 cycles of 28 days; treatment with temozolomide (1 dose-escalation to 200 mg/m^2^ if no toxicity in cycle 1), ascorbate (2 times per week, dose-escalation until 20 mM plasma concentration, around ~85 g infusion).	Ascorbate with radiation and temozolomide	Radiation phase toxicity: Grade 2 and 3 fatigue and nausea; grade 2 infection; grade 3 vomiting Adjuvant phase toxicity: grade 2 fatigue and nausea; grade 1 vomiting; grade 3 leukopenia; and grade 3 neutropenia.	Progression-free survival 13.3 months; average overall survival 21.5 months.
[30] Phase II study	14	Advanced stage non-small cell lung cancer	Single Arm	1 cycle is 21 days; IV carboplatin (AUC 6, 4 cycles), IV paclitaxel (200 mg/m^2^, 4 cycles), IV pharmacological ascorbate (two 75 g infusions per week, up to 4 cycles)	Carboplatin, paclitaxel, and ascorbate	No grade 3 or 4 toxicities related to ascorbate	Imaging confirmed partial responses to therapy (*n* = 4), stable disease (*n* = 9), disease progression (*n* = 1)
[65]	14	Locally advanced or metastatic prostate cancer	Single Arm	Phase I: Escalating dose of IVC from 25 g to 100 g and gemcitabine alone at 1000 mg/m^2^ (week 3) with a few patients receiving reduced doses and gemcitabine with IVC (week 4) Phase IIa: no gemcitabine for 1 week and then continuous treatment of gemcitabine until disease progression or unacceptable toxicity and IVC 3 times per week	IVC and gemcitabine	Low toxicity; Increased thirst and nausea were caused by IVC	Patients experienced a mix of stable disease, partial response and disease progression.

Note: This table illustrates the nine clinical trials that evaluated intravenous ascorbate in combination with non-redox cycling chemotherapy agents.

**Table 3:** High dose IV ascorbate only—Phase I and II trials.

Reference	*n*	Cancer Type	Trial Design	IV AA Treatment Type and Frequency	Toxicity	Reported Outcomes/Conclusions
	**Phase I**					
[18]	24	Advanced cancer or hematologic malignancy	Single Arm	1.5 g/kg body weight three times weekly	No dose limiting adverse effects.	Two patients had unexpectedly stable disease.
	**Phase II**					
[66]	23	Castration-resistant prostate cancer	Single Arm	5 g during weekly week 1, 30 g weekly during week 2, and 60 g weekly during weeks 3–12	Multiple grade 3 events including hypertension and anemia; two patients experienced pulmonary embolism.	Adverse events were thought to be more likely related to disease progression than ascorbic acid.
[23]	11	Late stage terminal cancer patients	Single Arm	150–710 mg/kg/day for up to eight weeks	Two Grade 3 adverse events: one patient with a history of renal calculi developed a kidney stone after thirteen days of treatment and another patient experienced hypokalemia after six weeks of treatment.	One patient had stable disease and continued the treatment for forty-eight weeks Intravenous vitamin C was deemed relatively safe so long as the patient does not have a history of kidney stone formation.

Note: This table illustrates the three IV ascorbate-only trials evaluated in this review. These trials evaluated IV ascorbate as a single intervention.

**Table 4:** Upcoming and active interventional trials utilizing pharmacological IV ascorbate.

Phase	Trial Title	Trial Design	IV AA Treatment Type and Frequency	Interventions	Status	Enrollment	NCT Identifier
**Phase I**	Gemcitabine, Ascorbate, Radiation Therapy for Pancreatic Cancer	Single Arm	50 g–100 g during radiation therapy for 5–6 weeks; escalating dose based on tolerance	Ascorbate Gemcitabine Radiation Therapy	Ongoing, closed to accrual	16	NCT01852890
**Phase I**	High-Dose Ascorbate in Glioblastoma Multiforme	Single Arm	15 g–87.5 g by IV 3×/week for 12 weeks	Ascorbate Temozolomide Radiation Therapy	Ongoing, closed to accrual	13	NCT01752491
**Phase I**	High Dose Ascorbic Acid (AA) + Nanoparticle Paclitaxel Protein Bound + Cisplatin + Gemcitabine (AA NABPLAGEM) in Patients Who Have No Prior Therapy for Their Metastatic Pancreatic Cancer	Single Arm	No dosing information provided	Ascorbic Acid Nab-paclitaxel Cisplatin Gemcitabine	Ongoing, actively recruiting participants	36	NCT03410030
**Phase II**	High-dose Ascorbate for Pancreatic Cancer (PACMAN 2.1)	Single Arm	75 g by IV 3×/week for 4 weeks	Ascorbate Gemcitabine Nab-paclitaxel	Accrual began 28 May 2018	30	NCT02905578
**Phase II**	High-Dose Ascorbate in Stage IV Non-Small Cell Lung Cancer	Single Arm	75 g by IV 2×/week for up to 12 weeks	Ascorbic Acid Carboplatin Paclitaxel	Ongoing, actively recruiting participants	57	NCT02420314
**Phase II**	High-Dose Ascorbate in Glioblastoma Multiforme	Single Arm	87.5 g by IV 3×/week during radiation therapy After radiation ascorbate 2×/week	Temozolomide Ascorbic Acid Radiation Therapy	Ongoing, actively recruiting participants	90	NCT02344355
**Phase II**	Adding Ascorbate to Chemotherapy and Radiation Therapy for NSCLC (XACT-LUNG)	Single Arm	Concurrent phase: 75 g by IV 3×/week for up to 7 weeks Consolidation phase: 75 g by IV 2×/week for two cycles (42 days)	Paclitaxel Carboplatin Ascorbate Radiation Therapy	Ongoing, actively recruiting participants	46	NCT02905591
**Phase II**	Docetaxel with or Without Ascorbic Acid in Treating Patients with Metastatic Prostate Cancer	Randomized	1 g/kg 3× per week	Docetaxel Ascorbic Acid or Placebo	Ongoing, actively recruiting participants	69	NCT02516670
**Phase I/II**	Randomized Study to Evaluate the Role of Intravenous Ascorbic Acid Supplementation to Conventional Neoadjuvant Chemotherapy in Women with Breast Cancer	Randomized	1.5 g on day 1 followed by 0.75 g on day 2–4 at each chemotherapy cycle	Ascorbic Acid Placebo	Status Unknown	30	NCT03175341
**Phase I/II**	Evaluating the Safety and Tolerability of Vitamin C in Patients with Intermediate or High Risk Myelodysplastic Syndrome with TET2 Mutations	Single Arm	50 gm CIVI/24 h x 5 days every 4 week	Ascorbic acid	Accrual begins 26 June 2018	18	NCT03433781

Note: This table illustrates the current and upcoming trials listed on clinicaltrials.gov that are utilizing standard of care chemotherapeutics in combination with pharmacological IV ascorbate.

## Abbreviations

AA	Ascorbic Acid
AML	Acute Myeloid Leukemia
APL	Acute Promyelocytic Leukemia
As_2_O_3_	Arsenic Trioxide
CT	Computed Tomography
CTCAE	Common Terminology Criteria for Adverse Events
DHA	Dehydroascorbic acid
DNA	Deoxyribonucleic acid
GBM	Glioblastoma
GADPH	Glyceraldehyde 3-phosphate dehydrogenase
GSH	Glutathione
HIF	Hypoxia inducible factors
H_2_O_2_	Hydrogen Peroxide
IV	Intravenous
IVC	Intravenous vitamin C
mM	Millimolar
NSCLC	Non-small cell lung cancer
OS	Overall survival
PFS	Progression free survival
PHD	Prolyl-4-hydroxylase domain
ROS	Reactive oxygen species
TET	Ten eleven translocation
